# The sodium–glucose cotransporter 2 inhibitor ipragliflozin improves liver function and insulin resistance in Japanese patients with type 2 diabetes

**DOI:** 10.1038/s41598-022-05704-y

**Published:** 2022-02-03

**Authors:** Tsuyoshi Okura, Yohei Fujioka, Risa Nakamura, Sonoko Kitao, Yuichi Ito, Mari Anno, Kazuhisa Matsumoto, Kyoko Shoji, Kazuhiko Matsuzawa, Shoichiro Izawa, Hiroko Okura, Etsuko Ueta, Masahiko Kato, Takeshi Imamura, Shin-ichi Taniguchi, Kazuhiro Yamamoto

**Affiliations:** 1 Division of Cardiovascular Medicine, Endocrinology and Metabolism, Tottori University Faculty of Medicine, 36-1 Nishi-Cho, Yonago, Tottori 683-8504 Japan; 2 School of Health Science, Tottori University Faculty of Medicine, Yonago, Japan; 3 Division of Molecular Pharmacology, Tottori University Faculty of Medicine, Yonago, Japan; 4 Department of Regional Medicine, Tottori University Faculty of Medicine, Yonago, Japan

**Keywords:** Endocrinology, Medical research

## Abstract

Sodium–glucose cotransporter 2 inhibitor (SGLT2i) treatment is a therapeutic approach for type 2 diabetes mellitus (T2DM). Some reports have shown that SGLT2i treatment improves insulin resistance; however, few studies have evaluated insulin resistance by the glucose clamp method. Hepatic insulin clearance (HIC) is a new pathophysiological mechanism of T2DM. The effect of SGLT2i treatment on hepatic insulin clearance and insulin resistance is not well known. We investigated the effect of SGLT2i treatment on insulin resistance, insulin secretion, incretin levels, body composition, and hepatic insulin clearance. We conducted a meal tolerance test (MTT) and a hyperinsulinemic-euglycemic clamp test in 9 T2DM patients. Ipragliflozin (50 mg/day) was administered, and the MTT and clamp test were performed after 4 months. We calculated HIC as the postprandial C-peptide AUC-to-insulin AUC ratio. We also measured GLP-1, GIP, and glucagon levels during the MTT. Body weight and HbA1c were decreased, although not significantly, after 4 months of treatment. Postprandial glucose, fasting insulin and postprandial insulin were significantly decreased. Insulin resistance with the glucose clamp was not changed, but the HOMA-IR and insulin sensitivity indices were significantly improved. Incretin and glucagon levels were not changed. Hepatic insulin clearance was significantly increased, but whole-body insulin clearance was not changed. The FIB-4 index and fatty liver index were significantly reduced. The HOMA-beta and insulinogenic indices were not changed, but the C-peptide index was significantly increased. Although the number of patients was small, these results suggested that SGLT2i treatment improved liver function, decreased hepatic insulin resistance, and increased hepatic insulin clearance, despite the small weight reduction.

## Introduction

The main pathophysiology of type 2 diabetes mellitus (T2DM) is decreased insulin sensitivity and decreased insulin secretion ability^1^. Sodium–glucose cotransporter inhibitor treatment (SGLT2i) is an antidiabetic therapeutic approach that increases glucosuria, induces a glucose-lowering effect, and reduces body weight^2^. Some studies have reported that SGLT2i treatment improves insulin resistance. Dapagliflozin was found to improve muscle insulin resistance, as evaluated by the euglycemic hyperinsulinemic clamp technique, in an American population^3^. However, this study and other studies indicated that SGLT2i treatment increases endogenous glucose production and glucagon secretion. In one study performed by dual-tracer glucose administration combined with a mixed meal in a European population, SGLT2i treatment increased insulin sensitivity and the secretion of glucagon-like peptide-1 (GLP-1) but increased endogenous glucose production and glucagon secretion^4^. In another study, a glucose clamp test and an oral glucose tolerance test were performed, and SGLT2i treatment was found to improve insulin sensitivity in an American population^5^. These results suggest that SGLT2i treatment improves muscle insulin sensitivity but increases endogenous glucose production via glucagon secretion.

Hepatic insulin clearance (HIC) is a novel and important pathophysiological mechanism of hyperinsulinemia, metabolic syndrome, and T2DM; hepatic insulin clearance is decreased in these metabolic conditions^6^. One study reported that SGLT2i treatment increased insulin clearance after the meal tolerance test^7^. However, we previously reported that Japanese patients with poorly controlled T2DM showed increased hepatic insulin clearance^8^.

An American study showed that the SGLT2i canagliflozin improved liver insulin sensitivity and beta-cell function and increased hepatic insulin clearance, as evaluated by the glucose clamp method and a mixed meal tolerance test^9^. However, the pathophysiology of diabetes is very different between American and Asian people; Asian people exhibit a low insulin secretion ability and insulin resistance with mild obesity^10,11^. Therefore, we need an study of SGLT2i treatment in Asian individuals.

According to the above results, SGLT2i treatment affects insulin resistance, incretin levels, and hepatic insulin clearance; however, these effects were evaluated in different studies and, therefore, the complete mechanism in the real world, especially in Asian or Japanese people, is not well known. We evaluated the effect of SGLT2i treatment on insulin resistance and whole-body insulin clearance by the glucose clamp method, and we evaluated the effect on hepatic insulin clearance and beta-cell function by a meal tolerance test in Japanese individuals. We also measured the levels of incretins such as GLP-1 and glucose-dependent insulinotropic polypeptide (GIP), as well as glucagon levels, by a meal tolerance test. Here, we show that SGLT2i treatment improves hepatic insulin resistance, increases hepatic insulin clearance, and improves liver function in Japanese people.

## Materials and methods

### Subjects

Nine type 2 diabetes patients were enrolled in the study at Tottori University Hospital between 2014 and 2017. We diagnosed type 2 diabetes by the World Health Organization (WHO) criteria^12^. We excluded patients with malignancy, hepatitis B or C, liver cirrhosis, pancreatitis, renal dysfunction, or current use of diabetogenic medications such as corticosteroids from the study. All patients were of Japanese ethnicity. This study had a single-arm design. Ipragliflozin (50 mg/day) was administered after the meal tolerance test (MTT) and glucose clamp test, and the MTT and clamp test were performed after 4 months. Patients were instructed to drink water to prevent dehydration.

We performed this study according to the principles of the Declaration of Helsinki. The Ethics Committee of the Tottori University Faculty of Medicine approved this study on 30/05/2014 (approval number 2439). The clinical trial number was UMIN000014521.

Trial registration: UMIN, UMIN000014521, registered (09/07/2014)Retrospectively registered, https://center6.umin.ac.jp/cgi-open-bin/ctr_e/ctr_view.cgi?recptno=R000016186.

### Involvement of patients and the public

The first patient was enrolled on 3 June, 2014. The patient was recruited from the outpatient department of Tottori University Hospital. The research questions were developed by the Ethics Committee and provided to all of the patients using a procedure that was approved by the Ethics Committee. This study was a single-arm intervention study. We chose the outcomes of HOMA-IR index and glucose disposal rate as the markers of insulin resistance. All patients were recruited as the outpatient facility of Tottori University Hospital. We obtained informed consent from all of the patients using a procedure that was approved by the Ethics Committee.

### Meal tolerance test (MTT)

The patients visited our hospital after overnight fasting. The patients ate a test meal developed by the Japan Diabetes Society (JDS) (460 kcal, 50% carbohydrate, 15% protein, 35% fat, and 1.6 g of salt)^13^. Plasma glucose, serum insulin, and C-peptide were measured at 0, 30, 60, 120, and 180 min after consumption of the test meal. We used the glucose oxidase method for glucose measurement and chemiluminescent immunoassays for insulin and C-peptide measurement (human insulin and C-peptide chemiluminescent immunoassay kits; Kyowa Medix, Japan). HbA1c was measured by high-performance liquid chromatography. HbA1c levels were converted from percentage values to the International Federation of Clinical Chemistry values (mmol/mol), and an HbA1c converter from the National Institutes of Diabetes and Digestive and Kidney Diseases was used^14^.

Insulin resistance was assessed by using insulin resistance indices calculated as follows:^15,16^

$${\text{Homeostatic model assessment for insulin resistance }}\left( {{\text{HOMA-IR}}} \right)^{15} \, = \, \left\{ {{\text{fasting plasma glucose }}\left( {{\text{mmol}}/{\text{L}}} \right)} \right\} \, \times \, \left\{ {{\text{fasting plasma insulin }}\left( {{\text{pmol}}/{\text{L}}} \right)} \right\} \, /{ 135}.$$

$${\text{Insulin sensitivity index }}\left( {{\text{ISI}}} \right)^{16} \, = { 1}0,000 \, /{\text{square root}}\left[ {\left\{ {{\text{ fasting plasma glucose }}\left( {{\text{mmol}}/{\text{L}}} \right) \, \times {\text{ fasting plasma insulin }}\left( {{\text{pmol}}/{\text{L}}} \right)} \right\} \, \times \, \{ {\text{mean glucose }}\left( {{\text{mmol}}/{\text{L}}} \right) \, \times {\text{ mean insulin }}\left( {{\text{pmol}}/{\text{L}}} \right){\text{ during the MTT}}\} } \right].$$

Insulin secretion was assessed by using insulin secretion indices as follows:^6,15,17^

$${\text{Homeostasis model assessment for beta-cell function }}\left( {{\text{HOMA-beta}}} \right)^{15} \, = \, \left[ {{2}0\, \times \,\{ {\text{fasting plasma insulin }}\left( {{\text{pmol}}/{\text{L}}} \right)\} } \right] \, / \, \left[ {\left\{ {{\text{fasting plasma glucose }}\left( {{\text{mmol}}/{\text{L}}} \right)} \right\} \, - { 3}.{5}} \right].$$

 

$$\text{Insulinogenic index}^{6} = \, \left[ {\left\{ {{\text{plasma insulin }}\left( {{\text{pmol}}/{\text{L}}} \right){\text{ at 3}}0{\text{ min}}} \right\} \, {-} \, \{ {\text{plasma insulin }}\left( {{\text{pmol}}/{\text{L}}} \right){\text{ at }}0{\text{ min}}\} } \right] \, / \, \left[ {\left\{ {{\text{ plasma glucose }}\left( {{\text{mmol}}/{\text{L}}} \right){\text{ at 3}}0{\text{ min}}} \right\} \, {-} \, \{ {\text{plasma glucose }}\left( {{\text{mmol}}/{\text{L}}} \right){\text{ at }}0{\text{ min}}\} } \right]$$

$${\text{C-peptide index }}\left( {{\text{CPI}}} \right)^{17}\, = \, \left\{ {{\text{fasting C-peptide }}\left( {{\text{mmol}}/{\text{L}}} \right)} \right\} \, / \, \left\{ {{\text{fasting plasma glucose }}\left( {{\text{mmol}}/{\text{L}}} \right) \, \times { 1}00} \right\}$$

We calculated hepatic insulin clearance (HIC) as follows:

${\text{HIC }} = \, \left\{ {{\text{area under the curve }}\left( {{\text{AUC}}} \right){\text{ for Cpeptide}}, \, 0{-}{12}0{\text{ min }}} \right\}/\left\{ {{\text{AUC for insulin}}, \, 0{-}{12}0{\text{ min}}) \, } \right\}$^6^. The AUC was calculated using a trapezoidal method.

### GLP-1, GIP, and glucagon assays

We obtained serum samples by using blood collection tubes containing a DPP-4 inhibitor (BD™ P800, BD Japan, Japan). We performed ethanol and solid-phase extractions for the assay^18^. We measured intact GLP-1 levels with an ELISA kit (GLP-1 (active) ELISA, Merck Millipore, Germany). We also measured active GIP with an ELISA kit (Human GIP, Active form Assay Kit, IBL, Japan). We next measured glucagon levels with an ELISA kit (Glucagon ELISA kit, Mercodia, Sweden).

### Hyperinsulinemic-euglycemic clamp

The glucose clamp method was performed as we previously reported^8,19^. We performed the hyperinsulinemic-euglycemic clamp method to evaluate insulin sensitivity with an artificial endocrine pancreas (STG 55, Nikkiso, Japan). We performed the protocol with a priming constant insulin infusion (100 mU/m^2^/min), and we maintained the plasma glucose level at 5.2 mmol/L (95 mg/dL) during the clamp. The steady-state plasma insulin level was reached at 1200 pmol/L in T2DM patients, based on previous studies^20^. We measured the steady-state glucose infusion rate (GIR) from 90–120 min, and we defined the mean GIR during this period as the glucose disposal rate (GDR). The GDR is generally used to mainly reflect peripheral insulin sensitivity. We next calculated the M/I value by setting the M value equal to the GDR and the I value equal to the final insulin concentration during the clamp, i.e., the peripheral insulin sensitivity adjusted by the final insulin concentration^21^. Whole-body insulin clearance was calculated as the insulin infusion rate divided by the steady-state insulin concentration^21^.

### FIB-4 index

The liver fibrosis level was estimated by the ${\text{FIB-4 index}}:{\rm{ age }}(\left[ {{\rm{yr}}} \right]\times{\rm{AST }}\left[ {{\rm{U}}/{\rm{L}}} \right]){\rm{ }}/{\rm{ }}(\left( {{\rm{PLT }}\left[ {{\rm{1}}{0^{\rm{9}}}/{\rm{L}}} \right]} \right)\times{({\rm{ALT}}\left[ {{\rm{U}}/{\rm{L}}} \right])^{{\rm{1}}/{\rm{2}}}})$^22^.

### Fatty liver index (FLI)

The fatty liver index was estimated as follows:^23^

$$\begin{aligned}&{\text{Fatty liver index }}\left( {{\rm{FLI}}} \right)\, =\\& \,\left( {{{\rm{e}}^{0.{\rm{953}} \times {\rm{loge}}\left( {{\rm{triglycerides}}} \right) + 0.{\rm{139}} \times {\rm{BMI}} + 0.{\rm{718}} \times {\rm{loge}}\left( {{\rm{GGT}}} \right) + 0.0{\rm{53}} \times {\rm{waistcircumference}} - {\rm{15}}.{\rm{745}}}}} \right){\rm{ }}/{\rm{ }}\left( {{\rm{1}}\, + \,{{\rm{e}}^{0.{\rm{953}} \times {\rm{loge}}\left( {{\rm{triglycerides}}} \right) + 0.{\rm{139}} \times {\rm{BMI}} + 0.{\rm{718}} \times {\rm{loge}}\left( {{\rm{GGT}}} \right) + 0.0{\rm{53}} \times {\rm{waistcircumference}} - {\rm{15}}.{\rm{745}}}}} \right)\, \times \,100\end{aligned}$$

### Body composition

Body composition was analyzed with an Inbody 770 body composition analyzer (Inbody Japan, Tokyo, Japan). Body water, percent body fat, body fat mass, lean body mass, skeletal muscle mass, trunk fat mass, and bone mineral mass were measured.

### Statistical analysis

Data are expressed as the mean ± standard deviation values. We assessed differences in the mean values of clinical parameters before and after SGLT2i treatment by using the Mann–Whitney *U* test. We identified correlations between nonparametric clinical variables by using Spearman correlation analysis. Values of *P* < 0.05 were considered to be significant. PRISM8 software (GraphPad Software, San Diego, USA) was used for all analyses. We conducted a power analysis to compare the pretreatment and posttreatment HOMA-IR indices as a primary endpoint using an EZR calculator^24^.

### Ethical approval

This study was approved by the Ethics Committee of the Faculty of Medicine, Tottori University (approval number 2439). The clinical trial number is UMIN000014521, registered 30/05/2014.

## Results

**Figure 1:**
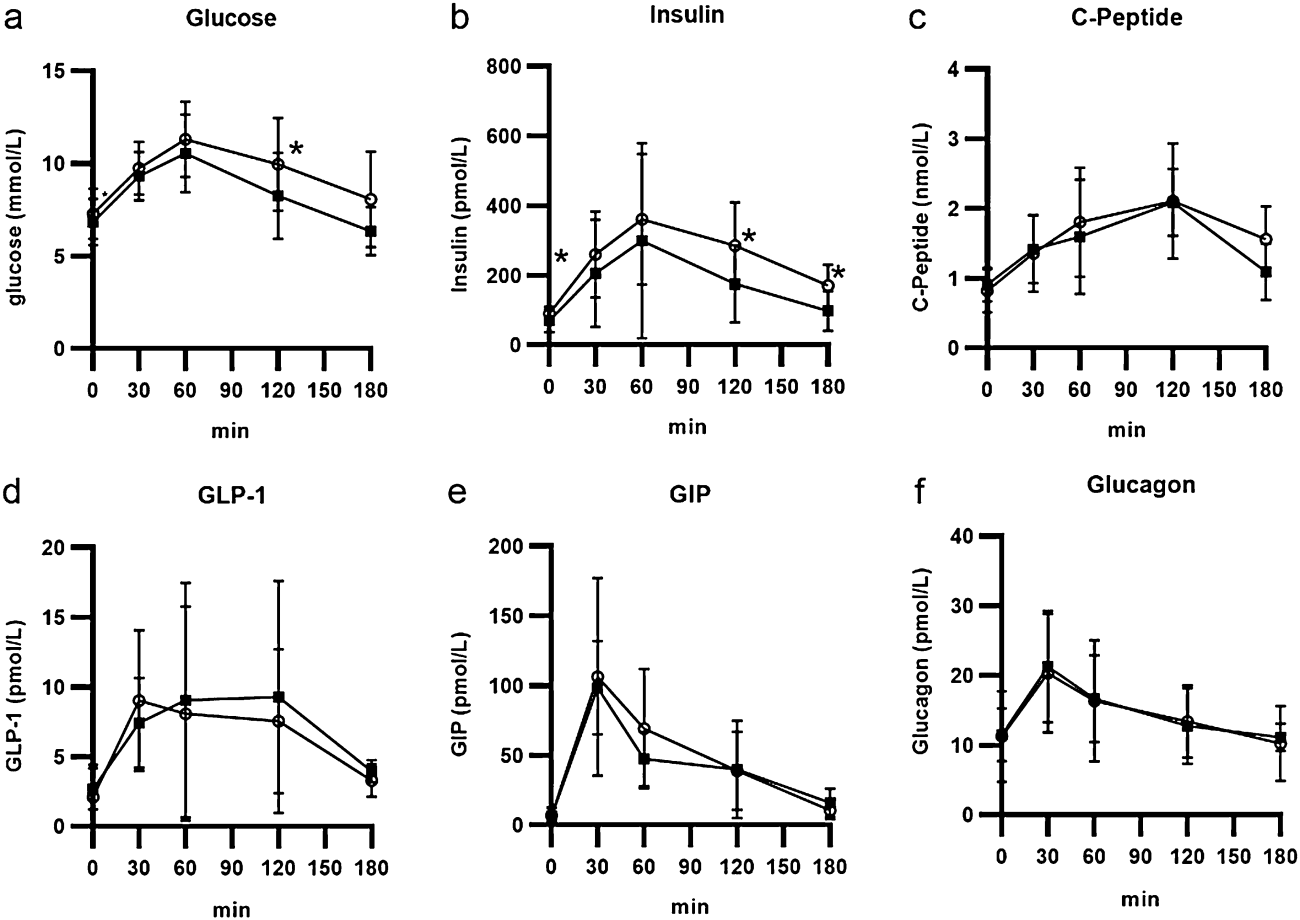
MTT Results. (**a**) shows glucose levels after the MTT, and (**b**) insulin, (**c**) C-peptide, (**d**) GLP-1, (**e**) GIP, and (**f**) glucagon levels are also shown. The white circles represent the pretreatment data, and the black squares indicate the posttreatment data. *, *p* < 0.05.

The mean age of the subjects was 54.1 ± 9.1 years, and there were 2 males and 7 females. The duration of diabetes was 5.0 ± 4.1 years. The participant characteristics are shown in Table 1. Seven patients were treated with only diet therapy, 2 patients took DPP4 inhibitors and alpha-glucosidase inhibitors, and 1 patient also took metformin. Table 1 shows the effects of SGLT2i treatment for 4 months. Body weight and HbA1c showed decreasing trends, but the trend was not significant. Body composition was not significantly changed. AST, the FIB-4 index, and the fatty liver index were significantly decreased. Postprandial glucose, fasting insulin and postprandial insulin were decreased. Figure 1 shows the MTT results. Glucose was significantly decreased 120 min after the MTT. The fasting, 60-min, and 120-min insulin levels were significantly decreased. C-peptide, GLP-1, GIP, and glucagon levels were not changed. Insulin resistance with the glucose clamp was not changed, but the HOMA-IR and insulin sensitivity indices were significantly improved. Incretin levels were not changed. Hepatic insulin clearance was significantly increased. Whole-body insulin clearance was not changed. The HOMA-beta and insulinogenic indices were not changed, but the CPI was significantly increased.

**Table 1:** Comparison between before and after SGLT2 treatment.

Parameter	Before	After	*P* value
Body weight (kg)	71.5 ± 9.1	70.2 ± 9.8	0.12
BMI (kg/m^2^)	28.2 ± 4.0	27.7 ± 4.0	0.11
Waist circumference (cm)	96.9 ± 9.2	96.5 ± 9.6	0.39
eGFR	86.2 ± 23.9	85.6 ± 24.3	0.84
AST (U/L)	28.1 ± 7.7	24.2 ± 8.7	< 0.05
ALT (U/L)	32.3 ± 14.6	28.5 ± 13.5	0.12
Gamma GTP (U/L)	42.5 ± 47.3	37.3 ± 41.0	0.11
FIB-4 index	0.92 ± 0.30	0.77 ± 0.32	< 0.05
Fatty liver index	59.8 ± 23.2	51.3 ± 27.9	< 0.05
HbA1c (%)	7.4 ± 1.1	6.8 ± 0.5	0.09
HbA1c (mmol/mol)	57.4 ± 12.0	50.8 ± 5.8	
**Insulin secretion**			
Fasting Plasma Glucose (mmol/L)	7.28 ± 1.35	6.83 ± 1.26	0.41
Postprandial Plasma Glucose 2 h (mmol/L)	9.94 ± 2.50	8.25 ± 2.30	< 0.05
Fasting Plasma insulin (pmol/L)	90.4 ± 20.9	69.9 ± 32.6	< 0.01
Postprandial Plasma insulin 2 h (pmol/L)	285.3 ± 122.9	175.1 ± 111.2	< 0.05
Glucose AUC (0–120 min)	362.4 ± 62.8	331.1 ± 61.5	0.17
Insulin AUC (0–120 min)	568.1 ± 250.9	370.9 ± 293.9	0.08
CPR AUC (0–120 min)	3.28 ± 1.33	2.90 ± 0.81	0.35
Hepatic insulin clearance (HIC)	6.03 ± 1.46	9.96 ± 4.26	< 0.05
HOMA-beta (%)	87.4 ± 32.7	78.5 ± 46.3	0.30
Insulinogenic Index	0.62 ± 0.25	0.63 ± 0.55	0.98
CPI (C-peptide index)	1.92 ± 0.79	2.25 ± 0.66	< 0.05
**Insulin resistance**			
HOMA-IR	4.86 ± 1.14	3.48 ± 1.53	< 0.05
Insulin sensitivity index	2.70 ± 0.81	4.67 ± 2.56	< 0.05
GDR (mg/kg/min)	5.52 ± 1.66	5.68 ± 2.63	0.77
M/I	4.97 ± 1.60	6.07 ± 4.38	0.65
Whole-body insulin clearance	1.37 ± 0.28	1.36 ± 0.39	0.95
**Incretins**			
GLP-1 AUC	14.8 ± 9.1	15.8 ± 11.2	0.41
GIP AUC	126.1 ± 75.6	106.3 ± 27.5	0.30
Glucagon AUC	13.3 ± 5.1	12.7 ± 5.4	0.44
**Body composition**			
Body water (L)	34.3 ± 4.2	33.7 ± 4.9	0.09
Percent body fat (%)	34.2 ± 7.6	34.5 ± 7.7	0.49
Body fat mass (kg)	24.7 ± 7.8	24.6 ± 7.9	0.72
Lean body mass (kg)	43.9 ± 5.5	43.2 ± 6.4	0.1
Skeletal muscle mass (kg)	25.6 ± 3.5	25.1 ± 4.1	0.1
Trunk fat mass (kg)	14.6 ± 5.5	15.2 ± 5.5	0.53
Bone mineral mass (kg)	2.6 ± 0.3	2.5 ± 0.4	0.34

Data are presented as the mean ± standard deviation values.

The comparison of parameters before and after treatment was performed using the Mann–Whitney *U* test. The chi-square test was used for categorical comparisons of sex data.

*ALT* alanine aminotransferase,* AST* aspartate aminotransferase,* AUC* area under the curve,* BMI* body mass index,* CPI* C-peptide index,* CPR* C-peptide immunoreactivity,* eGFR* estimated glomerular filtration rate,* FLI* fatty liver index,* GDR* glucose disposal rate,* GIP* glucose-dependent insulinotropic polypeptide,* GLP-1* glucagon-like peptide-1,* GGTP* γ-glutamyl transpeptidase,* HDL* high-density lipoprotein,* HIC* hepatic insulin clearance,* HOMA-beta* homeostatic model assessment for beta-cell function,* HOMA-IR* homeostatic model assessment for insulin resistance,* LDL* low-density lipoprotein,* SGLT2i* sodium–glucose cotransporter 2 inhibitor.

NS, not significant.

We conducted a power analysis to compare the pretreatment and posttreatment HOMA-IR index as a primary endpoint. The mean pretreatment HOMA-IR index was 4.86 ± 1.14, and the mean posttreatment HOMA-IR index was 3.48 ± 1.53. The difference between the mean pretreatment and posttreatment HOMA-IR indices was 1.38 ± 0.39. We conducted a power analysis of the statistical test that was used to compare the pre- and posttreatment HOMA-IR indices. Assuming that the difference in the means was 1.38, the SD was 0.39, alpha was 0.05, the 1-beta was 0.8, and R was 0.57, the estimated power was > 99.9%.

Table 2 shows the correlations between the changes in the HOMA-IR index and clinical parameters from pretreatment to after treatment. The change in hepatic insulin clearance was significantly correlated with the change in the HOMA-IR index. The change in waist circumference was also correlated with the change in the HOMA-IR index.

**Table 2:** Correlation coefficients between ΔHOMA-IR and clinical parameters.

Parameter	r	p
ΔBMI	0.251	NS
ΔWaist circumstance	0.667	< 0.05
ΔAST	− 0.336	NS
ΔALT	− 0.286	NS
ΔGGTP	0.109	NS
ΔFatty liver index	0.274	NS
ΔFIB-4 index	0.144	NS
ΔHDL	0.267	NS
ΔLDL	0.467	NS
ΔTriglyceride	0.443	NS
ΔHbA1c	− 0.235	NS
ΔeGFR	− 0.083	NS
ΔeGDR	− 0.517	NS
ΔM/I	− 0.233	NS
ΔWhole-body insulin clearance	− 0.548	NS
ΔHepatic insulin clearance (HIC)	− 0.600	< 0.005
ΔTotal body fat	0.217	NS
ΔGLP-1 AUC120	− 0.129	NS
ΔGIP AUC120	0.467	NS
ΔGlucagon AUC120	− 0.167	NS

Correlation coefficients were determined using the Spearman product-moment correlation analysis.

*ALT* alanine aminotransferase,* AST* aspartate aminotransferase,* AUC* area under the curve,* BMI* body mass index,* CPI* C-peptide index,* eGFR* estimated glomerular filtration rate,* GDR* glucose disposal rate,* GIP* glucose-dependent insulinotropic polypeptide,* GLP-1* glucagon-like peptide-1,* GGTP* γ-glutamyl transpeptidase,* HDL* high-density lipoprotein,* LDL* low-density lipoprotein.

*NS* not significant.

## Discussion

Our study indicated that treatment with the SGLT2i ipragliflozin improved the HOMA-IR index, liver function, and the C-peptide index but did not significantly change muscle insulin resistance, as evaluated by the glucose clamp method, nor did it alter body weight or body composition. SGLT2i increased hepatic insulin clearance but not whole-body insulin clearance. In a previous report, tofogliflozin was found to significantly improve insulin sensitivity and peripheral glucose uptake in Japanese patients with T2DM^25^. These improvements were significantly correlated with the reduction in body fat mass. Our study showed a tendency toward weight reduction, but the difference was not significant. This previous study included 16 patients and was a 12-week study; therefore, we consider our study to have a small sample number. Our study showed a trend toward a reduction in body water, but body fat mass did not change; these findings were different from those of the past study. However, the liver function parameters in our study were significantly improved, despite the small weight reduction, and we consider these points to be important. Dapagliflozin reduced liver fat but did not affect insulin sensitivity in liver tissue, muscle tissue, or adipocytes^26^. Another recent study also reported that SGLT2i treatment increased hepatic insulin clearance, as evaluated by an oral meal tolerance test^27^. A previous article reported that SGLT2i treatment increased GLP-1 and glucagon secretion^4^ and increased glucagon secretion from alpha cells^28^. We did not observe significant glucagon inhibition or changes in incretin levels by SGLT2i treatment, but this may depend on the small number of patients and the fact that 2 patients were already taking DPP4i drugs.

According to these results, SGLT2i decreased postprandial glucose levels and insulin levels. The HOMA-IR index mainly reflects liver insulin resistance, the glucose clamp method mainly reflects muscle insulin resistance, and the insulin sensitivity index reflects insulin sensitivity in both liver and muscle^29^. Although muscle insulin resistance, as evaluated by the glucose clamp method, was not improved, liver insulin resistance, as evaluated by the HOMA-IR index, was considered to be improved. Hyperinsulinemia was ameliorated after SGLT2i treatment, and this insulin-lowering effect might be induced by increased hepatic insulin clearance. These effects resulted in improved liver function. Finally, the C-peptide index was increased, which might mean that SGLT2i treatment has a protective effect on beta cells. Although further basic research in animals and cells is needed to understand the molecular mechanisms, our results contribute to understanding the effects of SGLT2i treatment. Asian individuals, who exhibit a low insulin secretion ability and insulin resistance with mild obesity, have a T2DM pathophysiology different from that of American and European individuals^10,11^. All patients in our study were Japanese; therefore, our results contribute to everyday clinical work in the Asian population. A recent Japanese study indicated that the SGLT2i tofogliflozin did not change whole-body insulin clearance^30^. According to these results, SGLT2i treatment mainly increases hepatic insulin clearance, not whole-body insulin clearance. We consider these results to be important for understanding the effects of SGLT2i treatment and the pathophysiology of T2DM.

Our study has some limitations. The major limitation is the very small number of patients (nine). However, glucose clamp tests and meal tolerance tests, including incretins, are very complicated and expensive studies. We need a larger study. We did not observe a significant reduction in HbA1c or body weight in this study, and 4 months might be a short treatment period. We evaluated insulin resistance by the glucose clamp method. In a previous report, the GDR was evaluated by subtracting the urinary glucose excreted from the total glucose administered^3^. We measured urinary glucose excretion in only 2 patients; therefore, we could not evaluate urinary glucose excretion, and we must consider this point. We did not evaluate hepatic insulin resistance by the tritium-glucose method because of the difficulty of using tritium-glucose in Japan. We evaluated hepatic insulin resistance by using only the HOMA-IR index, and further study is needed. We used the MTT to avoid hyperglycemia resulting from the OGTT in patients with severe diabetes. However, a previous study used the HIC obtained from the MTT; therefore, we think our method is acceptable^7^. Although our main purpose was to evaluate hepatic insulin clearance, whole-body and peripheral insulin clearance are also important, as previously reported^31^. Unfortunately, the steady-state C-peptide levels with the glucose clamp were not measured in this study, and we need to investigate the peripheral insulin clearance evaluated during the glucose clamp test. A recent article stated that the postload CPR/IRI molar ratio does not accurately evaluate hepatic insulin clearance^32^. We must be careful to evaluate our results. We evaluated beta-cell function by the C-peptide index. However, the C-peptide index is affected by renal function. We excluded patients with renal disorder as determined by Cr > 1.3 mg/ml, according to a past report^33^, and eGFR did not show a significant change; therefore, we consider our data to be reliable. Despite these limitations, we performed glucose clamp and meal tolerance tests and incretin measurement in Japanese patients with T2DM in a real-world setting. We consider that our study might contribute to our everyday clinical work, especially in the Asian population.

In conclusion, SGLT2i treatment improved liver function, decreased hepatic insulin resistance, and increased hepatic insulin clearance, despite the small weight reduction.

## Data Availability

No additional data are available.

## Abbreviations

ALT: Alanine aminotransferase
AST: Aspartate aminotransferase
AUC: Area under the curve
BMI: Body mass index
CPI: C-peptide index
CPR: C-peptide immunoreactivity
eGFR: Estimated glomerular filtration rate
FLI: Fatty liver index
GDR: Glucose disposal rate
GIP: Glucose-dependent insulinotropic polypeptide
GLP-1: Glucagon-like peptide-1
GGTP: γ-Glutamyl transpeptidase
HDL: High-density lipoprotein
HIC: Hepatic insulin clearance
HOMA-beta: Homeostatic model assessment for beta-cell function
HOMA-IR: Homeostatic model assessment for insulin resistance
LDL: Low-density lipoprotein
SGLT2i: Sodium–glucose cotransporter 2 inhibitor
